# Metacognitive awareness of cognitive problems in schizophrenia: exploring the role of symptoms and self-esteem

**DOI:** 10.1017/S0033291713001189

**Published:** 2013-06-05

**Authors:** M. Cella, S. Swan, E. Medin, C. Reeder, T. Wykes

**Affiliations:** Institute of Psychiatry, King's College London, UK

**Keywords:** Awareness, cognition, metacognition, schizophrenia, self-esteem

## Abstract

**Background:**

People with a diagnosis of schizophrenia have limited metacognitive awareness of their symptoms. This is also evident for cognitive difficulties when neuropsychological assessments and self-reports are compared. Unlike for delusions and hallucinations, little attention has been given to factors that may influence the mismatch between objective and subjectively reported cognitive problems. Symptom severity, and also self-esteem and social functioning, can have an impact on cognitive problem perception and help to explain the gap between objective and subjective cognitive assessments in psychosis.

**Method:**

One-hundred participants with a diagnosis of schizophrenia were recruited and assessed with a comprehensive neuropsychological battery, a measure of awareness of cognitive problems and measures of psychotic symptoms, social and behavioural functioning and self-esteem. Regression was used to investigate the influence of symptoms, social functioning and self-esteem, and patients with different levels of cognitive problem awareness were contrasted.

**Results:**

Simple correlation analysis replicated the lack of association between objective cognitive measures and metacognitive awareness of cognitive problems. However, the results of the regression analyses highlight that self-esteem and negative symptoms predict metacognitive awareness. When significant predictors were controlled, individuals with better awareness had more impaired working memory but higher IQ.

**Conclusions:**

Poor self-esteem and high negative symptoms are negatively associated with metacognitive awareness in people with schizophrenia. Interventions that aim to improve cognition should consider that cognitive problem reporting in people with schizophrenia correlates poorly with objective measures and is biased not only by symptoms but also by self-esteem. Future studies should explore the causal pathways using longitudinal designs.

## Introduction

Lack of symptom awareness is a common characteristic in people suffering from schizophrenia (Amador *et al.* 1993; David *et al.* 2012). Research on symptom awareness has traditionally focused on psychotic symptoms, with only more recent research exploring cognition (Aleman *et al.* 2006). Several reports have highlighted a mismatch between subjective assessments and outcomes from neuropsychological tests (Harvey *et al.* 2001; Stip *et al.* 2003; Medalia & Lim, 2004; Moritz *et al.* 2004; Keefe *et al.* 2006; Sanjuan *et al.* 2006). However, some studies have noted that a degree of awareness can be observed in some patients (e.g. Stip *et al.* 2003; Medalia *et al.* 2008). In the attempt to elucidate the discrepancies between subjective and performance-based assessments of cognitive problems, some contributions have suggested that general IQ may limit insight in schizophrenia (David *et al.* 1992, 1995; Rossell *et al.* 2003). Other studies suggest that cognitive shifting may be more strongly associated with cognitive symptom insight (Cuesta *et al.* 1995; Aleman *et al.* 2006). The relevance of psychopathology to cognitive symptom awareness has also been explored, but the findings are largely inconsistent (Ritsner & Blumenkrantz, 2007; De Hert *et al.* 2009). Despite the controversies about which particular domain is associated with cognitive symptoms awareness, there seems to be a consensus in the literature on the relevance of cognitive deficits awareness in people with schizophrenia.

Factors that contribute to poor awareness of cognitive problems are: gender (Cuffel *et al.* 1996; Mintz *et al.* 2003), age of onset (Lysaker & Bell, 1995) and lower education levels (Macpherson *et al.* 1996; Ritsner & Blumenkrantz, 2007), but the evidence is not conclusive (e.g. David *et al.* 1995; Goldberg *et al.* 2001). Unlike the other factors mentioned, cognition and low mood have been hypothesized to have a direct relationship with insight. Cognitive problems may, more intuitively, limit awareness simply by influencing the ability to retain and elaborate information (Cooke *et al.* 2007). Alternatively, some authors suggest that depression and poor self-esteem are associated with better insight because poor insight may function as a defence mechanism for depression (McGlashan & Carpenter, 1976). Studies conducted in individuals experiencing a manic episode also suggest that elated mood is associated with poor insight in the context of recovery (Michalakeas *et al.* 1994).

More recently, some researchers have begun to focus on the relationship between metacognition and symptoms insight (Lysaker *et al.* 2011*a*,*b*). The term metacognition is used to describe a person's reflection about their cognitive processes. Important aspects of metacognition are: monitoring (cognitive functioning evaluation), control (directing and evaluating cognitive and behavioural performance) and knowledge (understanding task difficulty and resources required). Awareness of cognitive problems can be thought of as a form of metacognitive knowledge that can effectively guide the deployment of cognitive resources to a specific task and can provide the necessary knowledge for individuals to access the relevant resources to perform at maximal efficiency (Flavell, 1979). Metacognition is traditionally measured with self-assessed measures. In the area of cognitive symptoms several assessment tools have been put forward, with some measures having a specific focus on metacognitive regulation (Beck *et al.* 2004; Koren *et al.* 2004) and others on metacognitive knowledge (Stip *et al.* 2003; Medalia & Thysen, 2008). In this study we have focused on metacognitive knowledge because of its relevance to treatment choices.

Despite the different focus, an assessment of cognitive problems from the point of view of the patient cannot disregard the relevance of factors such as symptoms, illness-related factors, and also self-related factors, as important elements that can influence reporting. Several studies have highlighted the importance of self-related factors and schemas in reflecting on cognitive symptoms (Cuffel *et al.* 1996; Ritsner & Blumenkrantz, 2007). In particular, a study by Lysaker *et al.* (2011*a*) and a review by David *et al.* (2012) advanced the possibility of a link between self-esteem and metacognition, with self-esteem being a possible biasing factor for the reporting of symptoms.

The investigation of factors likely to influence the reporting of cognitive problems is clinically relevant. In the context of behavioural interventions targeting cognitive difficulties, such as cognitive remediation, the subjective report of cognitive difficulties is crucial for goal shaping and therapy engagement (Huddy *et al.* 2012). Engagement in treatment is likely to be more positive if cognitive training is directed towards domains perceived as problematic. However, the mismatch between objective and subjective cognitive problems may lead to treatment that feels irrelevant to the client because it does not match the areas of difficulty perceived as problematic. Additionally, metacognition has been suggested as an important mediator linking cognitive improvement following cognitive remediation with functional outcomes (Wykes & Spaulding, 2011; Wykes *et al.* 2012); with this relationship being found not only in people with schizophrenia but also in individuals with traumatic brain injury (Cicerone *et al.* 2011).

Previous research has highlighted several factors that may influence symptom awareness. Positive and negative symptoms, level of function in everyday life and also self-esteem have been identified as potentially significant biasing factors for symptoms reporting (Lysaker *et al.* 2011*b*; Palmier-Claus *et al.* 2011; Cella *et al.* 2012; David *et al.* 2012). These factors have mostly been investigated in relation to psychotic features, in particular hallucination and delusion, with cognitive difficulties being largely neglected.

Given the previous literature, we hypothesized that self-esteem, psychotic symptoms and social functioning may play a relevant role in influencing awareness. Being poorly aware of symptoms and social functioning problems may lead individuals with schizophrenia to hold incongruent levels of self-esteem (e.g. where self-esteem is high in the presence of debilitating symptoms and poor social functioning). It is plausible to hypothesize that poor awareness of cognitive problems may be similarly dissociated with self-esteem, as the concept of self-esteem is inherently related to metacognition as it requires individuals to reflect upon their self-worth. No previous study has attempted to explore the role of self-esteem as a predictor of cognitive awareness. It is therefore possible that controlling for self-esteem may remove part of the self-reflective bias that could prevent patients' judgement from being closer to performance-based assessment and uncover associations in domains where the awareness bias is less pronounced.

The current study set out to examine how self-esteem, symptoms and level of function may influence awareness of cognitive problems. In line with previous reports, we expected that there would be a non-significant correlation between cognitive performance and cognitive problem awareness (e.g. Stip *et al.* 2003; Medalia & Lim, 2004; Medalia & Thysen, 2008). However, when controlling for symptoms, self-esteem and social functioning we expected that a relationship would emerge between subjective and objectively reported cognitive problems.

## Method

### Participants

Participants (*n* = 100) were recruited as part of a cognitive remediation study. Inclusion criteria were: age between 18 and 65 years, a DSM-IV diagnosis of schizophrenia or other psychotic disorder and cognitive impairment of 1 standard deviation (s.d.) below the population average in at least four out of eight cognitive domains. Potential participants were excluded if they had a history of learning disability/developmental disorder, a history of organic brain disorder or head trauma or a diagnosis of substance dependence, or if they required the use of an interpreter. Recruitment took place in clinical teams within the South London and Maudsley National Health Service (NHS) Foundation Trust and Sussex Partnership NHS Foundation Trust in the UK.

### Assessment

#### Subjective Scale to Investigate Cognition in Schizophrenia (SSTICS; Stip et al. 2003)

The SSTICS was used as a measure of awareness of cognitive problems. The questionnaire contains 21 items focusing on: memory, attention, executive functions and praxia. Each item, referring to how often a problem occurs, is rated on a five-point Likert scale ranging from ‘never’ to ‘very often’. A higher score suggests greater awareness of cognitive problems. The scale has good internal consistency (*α* = 0.86) and test–retest reliability (*r* = 0.8; Stip *et al.* 2003). For this study we used the SSTICS problem (SSTICS-P) score. The SSTICS-P score ranges from 0 to 21 and is calculated as the number of items endorsed at the ‘very often’ or ‘often’ level on the SSTICS. This score better captures the number of problems that patients are likely to mention in a consultation session and is therefore more clinically relevant.

#### Positive and Negative Syndrome Scale (PANSS; Kay et al. 1987)

The scores derived from this measure and used in the analysis were the positive and negative symptom subscales. A higher score on either scale indicates greater symptom severity. All the PANSS raters were trained by an experienced researcher; interview reliability was appropriate and assessed with independent ratings conducted on selected recorded interviews.

#### Rosenberg Self-Esteem Scale (RSES; Rosenberg, 1965)

The RSES was used to provide a measure of participants' self-esteem. A higher total score is indicative of higher self-esteem.

#### Social Behaviour Schedule (SBS; Wykes & Sturt, 1986)

The SBS was used as a measure of participants' social functioning and was completed by a member of each participant's care team on their observations of the participant's functioning over the past month. A higher total score suggests greater difficulty across social functioning domains.

#### Objective measures of cognition

Each participant completed a neuropsychological assessment including: the Rey Complex Figure (Meyers & Meyers, 1995), the Wisconsin Card Sorting Task (WCST; Heaton *et al.* 1993), the Hayling Sentence Completion Test (Burgess & Shallice, 1997) and the Digit Span, Digit Symbol Coding, Vocabulary and Block Design from the Wechsler Adult Intelligence Scale – Fourth Edition (WAIS-IV; Wechsler, 2008). Pre-morbid IQ was estimated with the Wechsler Test of Adult Reading (WTAR; Holdnack, 2001).

### Analysis

#### Data integrity

The Shapiro–Wilk test was used to assess variables' distribution normality and, where normality assumptions were violated, natural logarithmic transformation was performed prior to inclusion in parametric statistics (Sokal & Rohlf, 2012).

#### Relationship between subjective and objective assessments

Pearson correlation coefficients were calculated between the SSTICS-P score and the objective measures of cognition to assess the association. A linear, forced entry, regression model was used to specify the contribution of symptoms (i.e. positive and negative), social functioning and self-esteem to SSTICS-P scores. As participants were selected on the basis of their cognitive difficulties, high scores on the SSTICS-P indicate a better metacognitive awareness of problems and low scores indicate poorer metacognitive awareness. To define the relationships to metacognitive awareness more clearly, we defined two groups differing in their metacognitive awareness levels on the STICS-P score distribution: the SSTICS-HP (High Problem) included participants from the top quartile and the SSTICS-LP (Low Problem) included participants from the bottom quartile. The SSTICS-HP and STIPS-LP groups were contrasted using an ANCOVA with neuropsychological test performance entered as the dependent variable and factors significantly predicting the SSTICS-P score in the regression entered as covariates. For this analysis cognitive test performance was reported in standardized *z* scores based on this sample.

## Results

### Demographics

Of the 100 patients recruited for the study, 84 had a DSM-IV diagnosis of schizophrenia and 16 schizo-affective disorder. Table 1 presents demographic characteristics of the participants including pre-morbid IQ.

**Table 1.** Sociodemographic and clinical characteristics of the study population (n = 100)

Age, mean (s.d.)	39.28 (11.33)
Sex, % male	63
Marital status, %	
Single	82
Married	8
Other	10
Ethnicity, %	
White	41
Black	46
Asian	7
Mixed	6
Pre-morbid IQ, mean (s.d.)	93.72 (10.59)
Current FSIQ, mean (s.d.)	87.00 (13.69)
Years of education, mean (s.d.)	13.29 (2.39)
Employment status, %	
Paid employment	7
Voluntary work	18
Student	20
Unemployed	55
Weekly hours in employment, mean (s.d.)	
Paid employment (*n* = 8)	14.94 (11.78)
Voluntary employment (*n* = 23)	6.29 (8.93)
Years since first contact with mental health services, %	
< 1 year	3
1–5 years	16
5–10 years	20
> 10 years	61
Years since first admission to hospital, %	
Never admitted	8
< 1 year	1
1–5 years	18
5–10 years	20
> 10 years	53
Number of admissions in past 2 years, mean (s.d.)	0.62 (1.01)

FSIQ; Full-scale intelligence quotient; s.d., standard deviation.

### Data integrity

The SSTICS-P mean was 6.7 (s.d. = 5.4) with a variation range of 18 (minimum 0, maximum 18). Skewness was 0.37 and kurtosis was –1.2. The SSTICS-P internal consistency using Cronbach's *α* was 0.91.

### Metacognitive awareness and neuropsychological assessment

As expected, there were no significant correlations between the neuropsychological test scores and the SSTICS-P score (all *p* > 0.1) (see Fig. S1).

### Predictors of metacognitive awareness

With PANSS positive and negative subscales, SBS and RSES entered, the model explained 22% of the SSTICS-P variance (*F*_1,96_ = 5.68, *p* < 0.0001). The RSES score was the best predictor for the final model (*β* = –0.33, *p* < 0.0001). PANSS negative and total SBS scores made smaller contributions, with significance levels just below and approaching conventional significance threshold respectively (PANSS negative: *β* = –0.28, *p* < 0.046; SBS: *β* = 0.16, *p* = 0.056). PANSS positive did not contribute significantly (*β* = 0.03, *p* = 0.46).

### Comparing poor versus good awareness

The top quartile (SSTICS-HP) consisted of 24 participants and the bottom quartile (SSTICS-LP) consisted of 26 participants. As expected, the groups differed significantly on the number of problems identified, with mean problems for SSTICS-HP = 13.81 (s.d. = 2) and for SSTICS-LP = 0.31 (s.d. = 0.4) (*t*_48_ = –32.8, *p* < 0.0001). A multivariate ANCOVA was used to examine differences in mean population *z* scores between the two groups across the neuropsychological domains: working memory (WM); short-term memory recall (STR); long-term memory recall (LTR); attention (AT); processing speed (PS); executive function – set-shifting (EF-SS); executive function – inhibition (EF-IN); and full-scale IQ (FSIQ). The analysis controlled for self-esteem, PANSS negative score and also SBS total as this variable approached significance level.

**Fig. 1.**
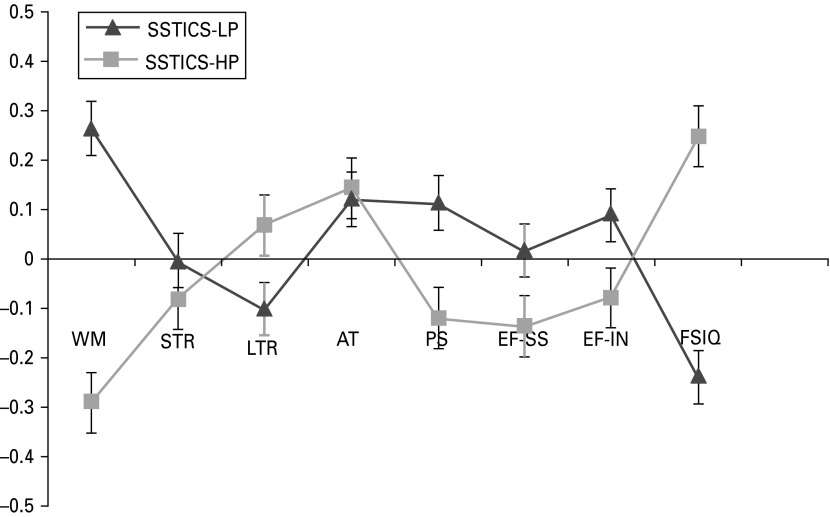
Neuropsychological profile (*z* scores with standard errors) of the Subjective Scale to Investigate Cognition in Schizophrenia Low Problem (SSTICS-LP) and SSTICS High Problem (SSTICS-HP) groups. WM, Working memory; STR, short-term recall; LTR, long-term recall; AT, attention; PS, procession speed; EF-SS, executive function, set-shifting; EF-IN, executive function, inhibition; FSIQ, full-scale IQ.

The two group profiles are shown in Fig. 1. The SSTICS-HP group had poorer working memory performance than the SSTICS-LP group (*F*_4,46_ = 4.51, *p* = 0.007, *η*^2^ = 0.23). IQ was higher in those reporting more problems (*F*_4,46_ = 2.9, *p* = 0.025, *η*^2^ = 0.26). *Post-hoc* ANOVA confirmed higher levels of self-esteem in the SSTICS-LP group (*F*_1,53_ = 4.06, *p* < 0.0001).

To further clarify the role of IQ in metacognitive awareness, an ANOVA was carried out to explore the role of IQ change, calculated by subtracting current IQ from pre-morbid IQ. The results show that IQ change did not differ between the SSTICS-HP and SSTICS-LP groups.

## Discussion

The aim of this study was to explore predictors of poor cognitive problem awareness in schizophrenia. Based on the literature we hypothesized that three factors would be important predictors: self-esteem, social functioning and symptom dimensions.

As hypothesized, the results replicated the mismatch between objective and subjective assessment of cognitive problems. Variance analysis showed that approximately a quarter of the variance in the subjective awareness of cognitive problems can be explained by self-esteem, negative symptoms and social functioning. The strongest predictor was self-esteem, with lower self-esteem values being predictive of better awareness. This result suggests that high self-esteem might only be preserved in the context of denial of cognitive difficulties, a notion that has been proposed in the context of insight and psychotic symptoms (David *et al.* 2012). This interpretation poses a challenge to interventions aiming to improve metacognitive awareness of problems because change in metacognitive levels may affect self-esteem negatively (Salvatore *et al.* 2012). This is, however, an empirically testable hypothesis that, as far as we know, has not been investigated. Wykes & Reeder (2005) and, more recently, Wykes & Spaulding (2011) proposed that more strategic approaches to cognitive remediation (rather than just practising tasks) may lead to improvements in metacognition that allow the transfer of cognitive gains to functional outcomes. The evidence so far is that self-esteem does not suffer following cognitive remediation and in some cases improves (Wykes *et al.* 1999) following strategic cognitive remediation. However, more specific relationships with measures of metacognition have not been explored. An alternative explanation could be that, after many years, patients become used to their cognitive problems, which, unlike psychotic symptoms, tend to fluctuate less. This may lead to a gradual repositioning of individuals' self-esteem levels to upper levels. Individuals who identified themselves as having more cognitive problems had worse working memory performance and better IQ. One explanation might be that awareness of cognitive problems differs from domain to domain and working memory problems may be easier and more obvious to recognize and report. Working memory problems are also marked in the pre-morbid stage of psychosis and feature heavily in family members, suggesting a genetic predisposition (Seidman *et al.* 2012). Longer experience with memory deficits, and also having experienced difficulties in a period prior to psychotic symptoms onset, may result in better awareness of this problem. This lends support to the hypothesis that awareness for cognitive symptoms in psychosis may be more accurate than that for psychotic symptoms because of their pre-psychotic nature and longer presence.

Alternatively, or additionally, the higher IQ scores in those reporting more working memory problems in the higher awareness group may indicate that a certain level of cognitive preservation influences reporting of cognitive symptoms. It seems unlikely that better general cognition would simply correlate with better awareness and many studies have confirmed that other crucial factors such as psychopathology, mood and demographic factors significantly influence this relationship (e.g. Macpherson *et al.* 1996; Rossell *et al.* 2003; David *et al.* 2012).

The relationship between self-esteem and metacognitive awareness is of importance for clinical practice and research. Although previous research has shown a positive association between cognitive improvements and self-esteem (e.g. Wykes *et al.* 1999) in the context of cognitive remediation, a more recent study has shown that this relationship depends largely on clients' awareness of cognitive state changes (Rose *et al.* 2008). Further evidence, produced in the context of improving general symptom awareness, suggests that higher levels of metacognitive awareness are associated with increased levels of hopelessness and poorer self-esteem (Lysaker *et al.* 2009). Despite the possible negative impact on self-esteem, improvements in metacognitive awareness can have a positive effect on therapeutic engagement. Therapy targeting areas perceived as not problematic by patients are unlikely to be perceived as important and worthy of effort. An improved understanding of cognitive problems, in the context of cognitive remediation, can facilitate the perceived relevance of cognitive tasks and their repetition. Hence, the negative effects on self-esteem should be considered as part of a comprehensive framework in which increased awareness may contribute to improving therapy engagement and perceived meaningfulness of the intervention and in turn reduce drop-outs. However, this is speculation and more research is needed to provide evidence in favour or against this. A recent study by Drake *et al.* (unpublished observations) suggests that cognitive remediation, purported to improve metacognitive awareness, made subsequent cognitive behavioural therapy (CBT) more efficient in terms of the number of sessions required for the same outcome (i.e. reduction in psychotic symptom).

The exploration of causation in terms of the associations between factors identified here requires alternative designs such as longitudinal or path analysis designs, and treatment studies would be clearly helpful in this context as change in factors following treatment would allow causal hypothesis testing. Direct subjective measures of metacognition are also needed. One method proposed by Koren *et al.* (2004) allows the measurement of decisions based (it is assumed) on a clear awareness of performance on the WCST. Although this experimental method has provided interesting data, anecdotal evidence reported by these authors suggests that the decisions made and the participants' subjective awareness might differ.

More generally, future research should investigate the importance of self-esteem as applied to cognitive rehabilitation treatments so that therapy programmes can limit self-esteem reduction in the context of awareness improvement. Evidence suggests that treatment effects are also influenced, to a significant extent, by therapists and it is likely that the extent of change in self-esteem resulting from improved metacognition may be associated with a therapist's experience (Medalia & Richardson, 2005; Huddy *et al.* 2012).

## Supplementary Material

Supplementary Material Supplementary information supplied by authors.

## Supplementary material

For supplementary material accompanying this paper visit http://dx.doi.org/10.1017/S0033291713001189.

## References

[ref1] Aleman A, Agrawal N, Morgan KD, David AS (2006). Insight in psychosis and neuropsychological function: meta-analysis. British Journal of Psychiatry 189, 204–212 16946354 10.1192/bjp.189.3.204

[ref2] Amador XF, Strauss DH, Yale SA, Gorman JM (1993). Assessment of insight in psychosis. American Journal of Psychiatry 150, 873–879 8494061 10.1176/ajp.150.6.873

[ref3] Beck AT, Baruch E, Balter JM, Steer RA, Warman DM (2004). A new instrument for measuring insight: the Beck Cognitive Insight Scale. Schizophrenia Research 68, 319–329 15099613 10.1016/S0920-9964(03)00189-0

[ref5] Burgess P, Shallice T (1997). The Hayling and Brixton Tests. Test Manual. Thames Valley Test Company: Bury St Edmunds, UK

[ref6] Cella M, Dymond S, Cooper A, Turnbull OH (2012). Cognitive decision modelling of emotion-based learning impairment in schizophrenia: the role of awareness. Psychiatry Research 196, 15–19 22349649 10.1016/j.psychres.2011.08.015

[ref7] Cicerone KD, Langenbahn DM, Braden C, Malec JF, Kalmar K, Fraas M, Felicetti T, Laatsch L, Harley JP, Bergquist T, Azulay J, Cantor J, Ashman T (2011). Evidence-based cognitive rehabilitation: updated review of the literature from 2003 through 2008. Archives of Physical Medicine and Rehabilitation 92, 519–530 21440699 10.1016/j.apmr.2010.11.015

[ref8] Cooke MA, Peters ER, Greenwood KE, Fisher PL, Kumari V, Kuipers E (2007). Insight in psychosis: influence of cognitive ability and self-esteem. British Journal of Psychiatry 191, 234–237 17766764 10.1192/bjp.bp.106.024653

[ref9] Cuesta MJ, Peralta V, Caro F, de Leon J (1995). Is poor insight in psychotic disorders associated with poor performance on the Wisconsin Card Sorting Test? American Journal of Psychiatry 152, 1380–1382 7653699 10.1176/ajp.152.9.1380

[ref10] Cuffel BJ, Alford J, Fischer EP, Owen RR (1996). Awareness of illness in schizophrenia and outpatient treatment adherence. Journal of Nervous and Mental Disease 184, 653–659 8955677 10.1097/00005053-199611000-00001

[ref11] David AS, Bedford N, Wiffen B, Gilleen J (2012). Failures of metacognition and lack of insight in neuropsychiatric disorders. Philosophical Transactions of the Royal Society of London. Series B, Biological Sciences 367, 1379–1390 10.1098/rstb.2012.0002 PMC3318769 22492754

[ref12] David A, Buchanan A, Reed A, Almeida O (1992). The assessment of insight in psychosis. British Journal of Psychiatry 161, 599–602 1422606 10.1192/bjp.161.5.599

[ref13] David A, van Os J, Jones P, Harvey I, Foerster A, Fahy T (1995). Insight and psychotic illness. Cross-sectional and longitudinal associations. British Journal of Psychiatry 167, 621–628 8564318 10.1192/bjp.167.5.621

[ref14] De Hert MA, Simon V, Vidovic D, Franic T, Wampers M, Peuskens J, van Winkel R (2009). Evaluation of the association between insight and symptoms in a large sample of patients with schizophrenia. European Psychiatry 24, 507–512 19540728 10.1016/j.eurpsy.2009.04.004

[ref15] Flavell JH (1979). Metacognition and cognitive monitoring: a new area of cognitive-developmental inquiry. American Psychologist 34, 906–911

[ref16] Goldberg RW, Green-Paden LD, Lehman AF, Gold JM (2001). Correlates of insight in serious mental illness. Journal of Nervous and Mental Disease 189, 137–145 11277349 10.1097/00005053-200103000-00001

[ref17] Harvey PD, Serper MR, White L, Parella MJ, McGurk SR, Moriarty PJ, Bowie C, Vadhan N, Friedman J, David KL (2001). The convergence of neuropsychological testing and clinical ratings of cognitive impairment in patients with schizophrenia. Comprehensive Psychiatry 42, 306–313 11458305 10.1053/comp.2001.24587a

[ref18] Heaton R, Chelune G, Talley J, Kay G, Curtiss G (1993). Wisconsin Card Sorting Test Manual – Revised and Expanded. Psychological Assessment Resources: Odessa, FL

[ref19] Holdnack HA (2001). Wechsler Test of Adult Reading: WTAR. Psychological Corporation: San Antonio, TX

[ref20] Huddy V, Reeder C, Kontis D, Wykes T, Stahl D (2012). The effect of working alliance on adherence and outcome in cognitive remediation therapy. Journal of Nervous and Mental Disease 200, 614–619 22759940 10.1097/NMD.0b013e31825bfc31

[ref21] Kay SR, Fiszbein A, Opler LA (1987). The positive and negative syndrome scale (PANSS) for schizophrenia. Schizophrenia Bulletin 13, 261–276 3616518 10.1093/schbul/13.2.261

[ref22] Keefe RSE, Poe M, Walker TM, Kang JW, Harvey PD (2006). The Schizophrenia Cognitive Rating Scale: an interview-based assessment and its relationship to cognition, real-world functioning, and functional capacity. American Journal of Psychiatry 163, 426–432 16513863 10.1176/appi.ajp.163.3.426

[ref23] Koren D, Seidman LJ, Poyurovsky M, Goldsmith M, Viksman P, Zichel S, Klein E (2004). The neuropsychological basis of insight in first-episode schizophrenia: a pilot metacognitive study. Schizophrenia Research 70, 195–202 15329296 10.1016/j.schres.2004.02.004

[ref24] Lysaker P, Bell M (1995). Work rehabilitation and improvements in insight in schizophrenia. Journal of Nervous and Mental Disease 183, 103–106 7844571 10.1097/00005053-199502000-00007

[ref25] Lysaker PH, Buck KD, Salvatore G, Popolo R, Dimaggio G (2009). Lack of awareness of illness in schizophrenia: conceptualizations, correlates and treatment approaches. Expert Review of Neurotherapeutics 9, 1035–1043 19589052 10.1586/ern.09.55

[ref26] Lysaker PH, Dimaggio G, Buck KD, Callaway SS, Salvatore G, Carcione A, Nicolò G, Stanghellini G (2011 *a*). Poor insight in schizophrenia: links between different forms of metacognition with awareness of symptoms, treatment need, and consequences of illness. Comprehensive Psychiatry 52, 253–260 21497218 10.1016/j.comppsych.2010.07.007

[ref27] Lysaker PH, Erickson M, Ringer J, Buck KD, Semerari A, Carcione A, Dimaggio G (2011 *b*). Metacognition in schizophrenia: the relationship of mastery to coping, insight, self-esteem, social anxiety, and various facets of neurocognition. British Journal of Clinical Psychology 50, 412–424 22003950 10.1111/j.2044-8260.2010.02003.x

[ref28] Macpherson R, Jerrom B, Hughes A (1996). Relationship between insight, educational background and cognition in schizophrenia. British Journal of Psychiatry 168, 718–722 8773814 10.1192/bjp.168.6.718

[ref29] McGlashan TH, Carpenter WT (1976). Postpsychotic depression in schizophrenia. Archives of General Psychiatry 33, 231–239 766720 10.1001/archpsyc.1976.01770020065011

[ref30] Medalia A, Lim R (2004). Self-awareness of cognitive functioning in schizophrenia. Schizophrenia Research 71, 331–338 15474903 10.1016/j.schres.2004.03.003

[ref31] Medalia A, Richardson R (2005). What predicts a good response to cognitive remediation interventions? Schizophrenia Bulletin 31, 942–953 16120830 10.1093/schbul/sbi045

[ref32] Medalia A, Thysen J (2008). Insight into neurocognitive dysfunction in schizophrenia. Schizophrenia Bulletin 34, 1221–1230 18199632 10.1093/schbul/sbm144 PMC2632502

[ref33] Medalia A, Thysen J, Freilich B (2008). Do people with schizophrenia who have objective cognitive impairment identify cognitive deficits on a self report measure? Schizophrenia Research 105, 156–164 18718740 10.1016/j.schres.2008.07.007

[ref34] Meyers J, Meyers K (1995). Rey Complex Figure and Recognition Trial: Professional Manual. Psychological Assessment Resources: Odessa, FL

[ref35] Michalakeas AS, Skoutas C, Charalambous A, Peristeris A, Marinos V, Keramari E, Theologou A (1994). Insight in schizophrenia and mood disorders and its relation to psychopathology. Acta Psychiatrica Scandinavica 90, 46–49 7976449 10.1111/j.1600-0447.1994.tb01554.x

[ref36] Mintz AR, Dobson KS, Romney DM (2003). Insight in schizophrenia: a meta-analysis. Schizophrenia Research 61, 75–88 12648738 10.1016/s0920-9964(02)00316-x

[ref38] Moritz S, Ferahli S, Naber D (2004). Memory and attention performance in psychiatric patients: lack of correspondence between clinician-rated and patient-rated function with neuropsychological test results. Journal of the International Neuropsychological Society 10, 623–633 15327740 10.1017/S1355617704104153

[ref39] Palmier-Claus JE, Dunn G, Morrison AP, Lewis SW (2011). The role of metacognitive beliefs in stress sensitisation, self-esteem variability, and the generation of paranoia. Cognitive Neuropsychiatry 16, 530–546 22098083 10.1080/13546805.2011.561583

[ref40] Ritsner MS, Blumenkrantz H (2007). Predicting domain-specific insight of schizophrenia patients from symptomatology, multiple neurocognitive functions, and personality related traits. Psychiatry Research 149, 59–69 17137634 10.1016/j.psychres.2006.01.002

[ref41] Rose D, Wykes T, Farrier D, Dolan A-M, Sporle T, Bogner D (2008). What do clients think of cognitive remediation therapy? A consumer-led investigation of satisfaction and side effects. American Journal of Psychiatric Rehabilitation 11, 181–204

[ref42] Rosenberg M (1965). Society and the Adolescent Self-Image. Princeton University Press: Princeton, NJ

[ref43] Rossell SL, Coakes J, Shapleske J, Woodruff PW, David AS (2003). Insight: its relationship with cognitive function, brain volume and symptoms in schizophrenia. Psychological Medicine 33, 111–119 12537042 10.1017/s0033291702006803

[ref44] Salvatore G, Lysaker PH, Gumley A, Popolo R, Mari J, Dimaggio G (2012). Out of illness experience: metacognition-oriented therapy for promoting self-awareness in individuals with psychosis. American Journal of Psychotherapy 66, 85–106 22523795 10.1176/appi.psychotherapy.2012.66.1.85

[ref45] Sanjuan J, Aguilar EJ, Olivares JM, Ros S, Montejo AL, Mayoral F, Gonzales-Torres MA, Bousono M (2006). Subjective perception of cognitive deficit in psychotic patients. Journal of Nervous and Mental Diseases 194, 58–60 10.1097/01.nmd.0000195308.62107.31 16462557

[ref46] Seidman LJ, Meyer EC, Giuliano AJ, Breiter HC, Goldstein JM, Kremen WS, Thermenos HW, Toomey R, Stone WS, Tsuang MT, Faraone SV (2012). Auditory working memory impairments in individuals at familial high risk for schizophrenia. Neuropsychology 26, 288–303 22563872 10.1037/a0027970 PMC3539430

[ref47] Sokal RR, Rohlf FJ (2012). Biometry: The Principles and Practice of Statistics in Biological Research, 4th edn. W. H. Freeman and Company: New York

[ref48] Stip E, Caron J, Renaud S, Pampoulova T, Lecomte Y (2003). Exploring cognitive complaints in schizophrenia: the Subjective Scale to Investigate Cognition in Schizophrenia. Comprehensive Psychiatry 44, 331–340 12923712 10.1016/S0010-440X(03)00086-5

[ref49] Wechsler D (2008). Wechsler Adult Intelligence Scale – Fourth Edition. Pearson: San Antonio, TX

[ref50] Wykes T, Reeder C (2005). Cognitive Remediation Therapy for Schizophrenia. Routledge: Sussex, UK

[ref51] Wykes T, Reeder C, Corner J, Williams C, Everitt B (1999). The effects of neurocognitive remediation on executive processing in patients with schizophrenia. Schizophrenia Bulletin 25, 291–307 10416732 10.1093/oxfordjournals.schbul.a033379

[ref52] Wykes T, Reeder C, Huddy V, Taylor R, Wood H, Ghirasim N, Kontis D, Landau S (2012). Developing models of how cognitive improvements change functioning: mediation, moderation and moderated mediation. Schizophrenia Research 138, 88–93 22503640 10.1016/j.schres.2012.03.020 PMC3405533

[ref53] Wykes T, Spaulding WD (2011). Thinking about the future cognitive remediation therapy – what works and could we do better? Schizophrenia Bulletin 37, 80–90 10.1093/schbul/sbr064 PMC3160118 21860051

[ref54] Wykes T, Sturt E (1986). The measurement of social behaviour in psychiatric patients: an assessment of the reliability and validity of the SBS schedule. British Journal of Psychiatry 148, 1–11 3082403 10.1192/bjp.148.1.1

