# Supplementary figures and images for: Metacognitive awareness of cognitive problems in schizophrenia: exploring the role of symptoms and self-esteem
Source: Psychol Med. 2013 Jun 5;44(3):469–76. doi: 10.1017/S0033291713001189 (PMC3880064; doi:10.1017/S0033291713001189)

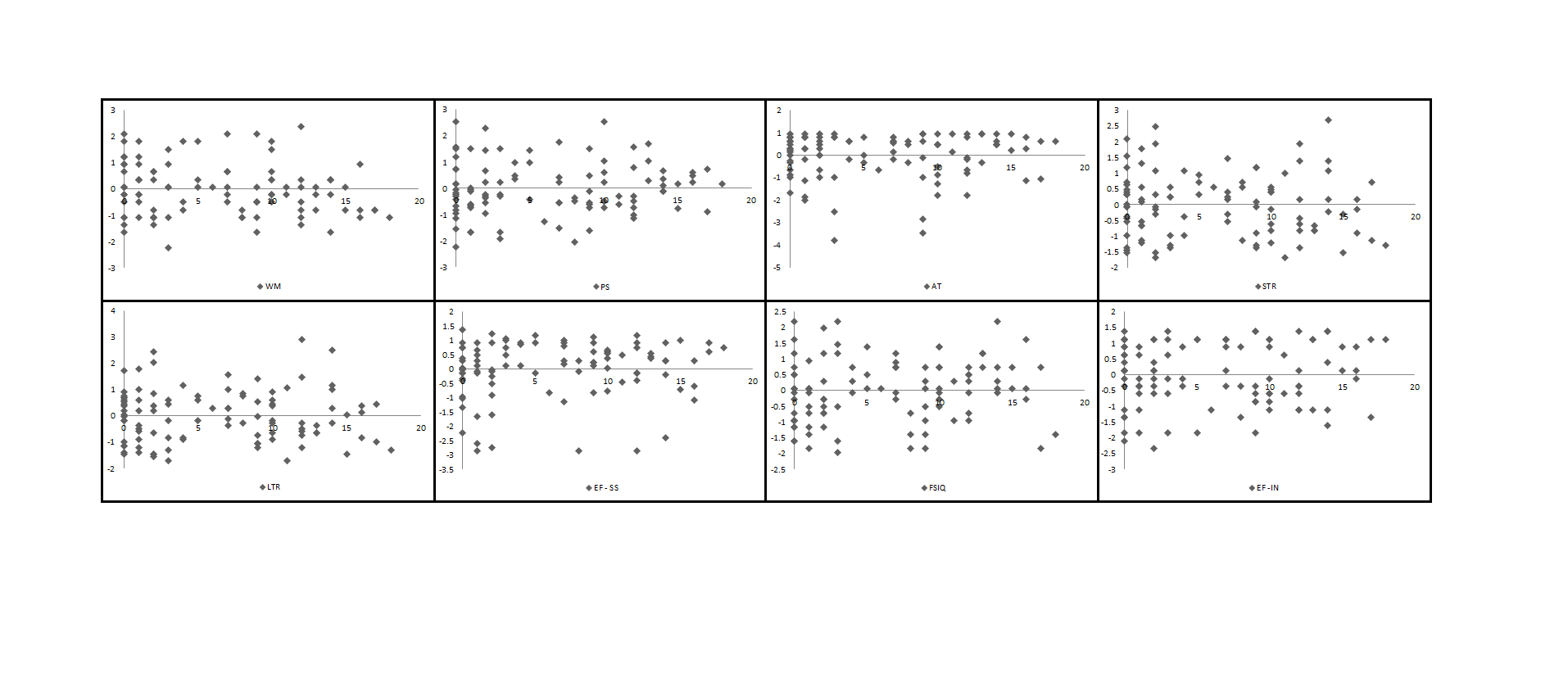

Supplement: Supplementary Material — Supplementary information supplied by authors. [file S0033291713001189sup001.tif]
